# Stress Detection of Conical Frustum Windows in Submersibles Based on Polarization Imaging

**DOI:** 10.3390/s22062282

**Published:** 2022-03-16

**Authors:** Hening Li, Ran Liao, Hailong Zhang, Guoliang Ma, Zhiming Guo, Haibo Tu, Yan Chen, Hui Ma

**Affiliations:** 1 Institute for Ocean Engineering, Shenzhen International Graduate School, Tsinghua University, Shenzhen 518055, China; lhn19@mails.tsinghua.edu.cn (H.L.); gzm20@mails.tsinghua.edu.cn (Z.G.); 202071210@yangtzeu.edu.cn (H.T.); 201971160@yangtzeu.edu.cn (Y.C.); 2 Guangdong Research Center of Polarization Imaging and Measurement Engineering Technology, Shenzhen International Graduate School, Tsinghua University, Shenzhen 518055, China; mahui@tsinghua.edu.cn; 3 Department of Biomedical Engineering, Tsinghua University, Beijing 100084, China; 4 Institute of Deep-Sea Science and Engineering, Chinese Academy of Sciences, Sanya 572000, China; zhanghailong@idsse.ac.cn (H.Z.); magl@idsse.ac.cn (G.M.); 5 Department of Physics, Yangtze University, Jingzhou 434100, China

**Keywords:** conical frustum window, PMMA, polarization imaging, stress detection

## Abstract

Stress detection of the conical frustum window is a very important issue to ensure the safety of deep manned submersibles. In this paper, we propose a method based on polarization imaging to evaluate the stress accumulation and recovery in the conical frustum window. An experimental setup of Mueller matrix polarimetry is built, and the samples are made by referring to the typical conical frustum windows in submersibles. By pressurizing different pressures on the samples, we can find the changes of their Mueller matrix images and further derived polarization parameters. The results show that the polarization parameters can characterize the stress transfer process and the elastic–plastic transformation process of the window under different pressurization pressures. We also use a two-layered wave plate model to simulate the stress distribution in the window, which reveals different performances of the former and latter layers of the window under pressurization. Finally, we use a finite element model to simulate and understand some of the above experimental results. This proposed method is expected to provide new possibilities for monitoring the window stress and further ensure the safety of deep manned submersibles.

## 1. Introduction

The pressure hull is an important part of a deep manned submersible, which is mainly composed of a pressure shell made of high-strength titanium alloy and observation windows made of polymethyl methacrylate (PMMA) material [1]. The observation window is a key component as it can provide a channel for scientists to observe the external sea conditions. Meanwhile, it is also an important pressure-bearing component, and stress accumulation will occur in the window due to the high external pressure in the diving process of the submersible [2,3]. The stress distribution inside the observation window affects its pressure resistance. The stress release degree of the observation window after the submersible surfaces is an important factor affecting the use frequency of the submersible [4]. Cracks in the window will threaten human safety inside the submersible. Therefore, it is of great significance for the safety and stability of the submersible to monitor the internal stress accumulation of the observation window and provide an evaluation standard of the window stress release degree in the recovery stage.

Many scientists have simulated the internal stress of the observation window by finite element analysis. Du et al. [5] studied the stress and deformation characteristics of the conical viewport window with the flange. Zhou et al. [6] showed the creep behavior of thick PMMA immersed in a liquid scintillator at eight stress levels. Arnold et al. [7] built predictive models for the creep behavior of PMMA, which match the experimental results. Pranesh et al. [8] showed several models of a viewport window to reduce the internal stress, which can reduce the corner stress by selecting a specific fillet radius. Liu et al. [9] identified unknown viscoelastic parameters and accurately analyzed the deep-water damage by comparing experiments with finite element analysis. Wang et al. [10] analyzed the time-deformation behavior of the observation window using the viscoelastic model. There are also several practical stress measurement methods, each of which has its own advantages and disadvantages. For example, the strain gauge method [11,12] can accurately measure the stress but only characterizes the surface stress and strain, while the ultrasonic method [13,14] can measure the internal stress of the bulk sample but sometimes causes damage to the sample, and the laser speckle method [15,16] can simultaneously measure the large area of the sample surface but requires the window surface to be roughened. Considering that stress accumulation occurs inside the window during diving, an in situ detection method for the submarine is still desired. To our knowledge, the development of a nondestructive, in situ and real-time stress detection method is currently challenging for the scientific community.

Polarization is the inherent property of light. Polarimetric techniques have been demonstrated to provide multidimensional parameters, which are sensitive to the microstructure of the samples [17]. Recently, polarimetric techniques have been used in biomedical therapy [18,19], marine particle probing [20,21], aerosol monitoring [22,23], etc.

Usually, we use the Stokes vector $S={\left(I,Q,U,V\right)}^{T}$ to describe the polarization state of light. When a beam of polarized light passes through the sample, the sample’s polarization property, always represented as a 4 × 4 Mueller matrix, will affect the polarization state of the incident light. Mathematically, we can obtain Equation (1) to describe the transformation process, where ${\left(I,\;Q,\;U,\;V\right)}^{T}$ is the Stokes vector of the incident light and ${\left(I\prime ,\;Q\prime ,\;U\prime ,\;V\prime \right)}^{T}$ is the Stokes vector of the output light.

(1) $$\left(\begin{array}{c} I^{\prime }\\ Q^{\prime }\\ U^{\prime }\\ V^{\prime } \end{array}\right)=\left(\begin{array}{cccc} m_{11} & m_{12} & m_{13} & m_{14}\\ m_{21} & m_{22} & m_{23} & m_{24}\\ m_{31} & m_{32} & m_{33} & m_{34}\\ m_{41} & m_{42} & m_{43} & m_{44} \end{array}\right)\left(\begin{array}{c} I\\ Q\\ U\\ V \end{array}\right)$$

At present, there are many polarization systems to measure the Mueller matrix of samples. The classical methods of rotating the optical polarization components, such as the polarizer and the quarter-wave plate, have been extensively used in measuring the Mueller matrix of biomedical tissues and integrated electronic chips [24]. However, reducing the rotational components can often improve the acquisition speed, measurement accuracy and stability of the polarimetry system. Recently, liquid crystal modulators [25,26] and photoelastic crystal devices [27,28] tend to gradually replace rotational components. In the meantime, new types of polarimeters are emerging to directly measure the polarization states of light in one or two dimensions. For example, the division of a focal plane (DoFP) polarimeter is capable of measuring the linear polarization states in a single shot, which consists of a common CCD sensor with a pixelated micro-polarizer array (MPA) in front of it. Several recent studies have used the DoFP polarimeter to measure the polarization properties of biological tissues [29,30]. Compared with traditional Mueller matrix polarimetry, it effectively improves the measurement speed and enhances the system’s stability.

In this study, polarization imaging is used to measure the stress and strain of the conical frustum window during pressurization and recovery stages. We firstly build an experimental setup to measure the Mueller matrix of the samples referring to the typical conical frustum window in submersibles. In the experiment, a controllable jack is used to pressurize the sample step by step, and the Mueller matrix of the sample is measured at each pressure level. The results demonstrate that the polarization parameters derived from the Mueller matrix can characterize the stress accumulation and release process of the sample in both the pressurization and recovery stages and provide effective indicators for the elastic–plastic transformation of the internal structure. Moreover, a two-layered wave plate simulation is proposed to describe the stress distribution in the sample, which is consistent with the results of finite element analysis. The results in this work indicate that polarization imaging can effectively detect the stress of the samples referring to the typical conical frustum window in submersibles, and this implies the possibility of monitoring the stress distribution in the conical frustum window quantitatively in the future.

## 2. Materials and Methods

### 2.1. Material

**Figure 1:**
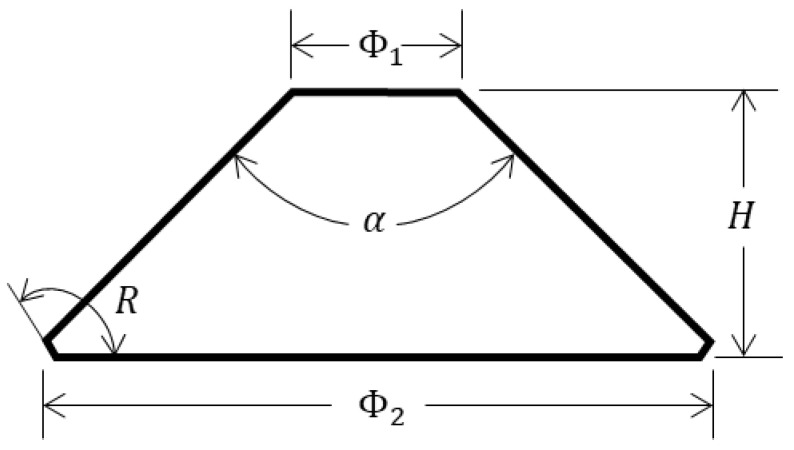
Structural parameters of sample.

Since Piccard first proposed a conical observation window in marine engineering in the 1950s, the conical observation window has been widely used in deep-sea submersibles. For convenience, we refer to the typical conical observation window of submersibles and designed the samples to be made of PMMA material to carry out the pressure experiment. In order to facilitate a description in this context, the large face of the sample is called the former face and the small face is called the latter face, according to the sequence of light passing through the sample in the experiment. The structure of the sample (Tiemao Glass, China) is shown in Figure 1 with the diameter of the latter face $\Phi_{1}=20\;\mathrm{mm}$, the diameter of the former face $\Phi_{2}=80.5\;\mathrm{mm}$ (to follow the direction of light passing through the sample), the height H=32.3 mm, the former face chamfer angle R=115°, and the cone Angle α=90°. The mechanical parameters of the sample are shown in Table 1.

### 2.2. Experiment Setup

**Figure 2:**
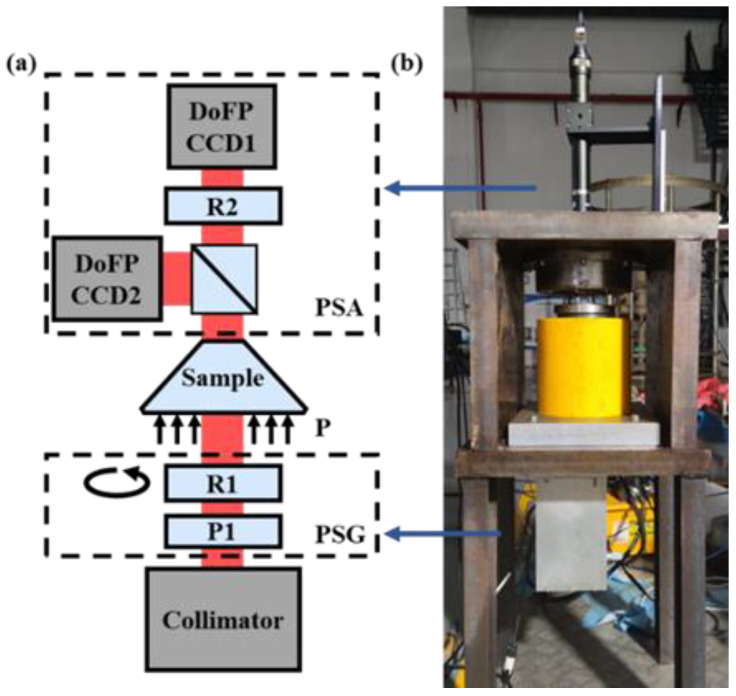
Schematic configuration (**a**) and photograph (**b**) of the experiment setup. P1, polarizer; R1 and R2, achromatic quarter-wave plates.

The experimental setup, as shown in Figure 2, was built to measure the Mueller matrix images of the sample when pressurizing different pressures on it. The setup consisted of a light collimator, a polarization state generator (PSG), a polarization state analyzer (PSA), and a pressurization device. In the light collimator, the light emitted from an LED lamp with the 630 nm central wavelength and 10 nm bandwidth was collimated by an optical system to finally form a parallel light beam whose transversal homogeneity was larger than 93%. The beam diameter was 20 mm, which can completely cover the latter face of the sample. The light beam successively passed PSG, the sample, and finally PSA, and the light beam’s polarization states were modulated by PSG, while its Stokes vector image after passing the sample was detected by PSA. A jack with a maximum working pressure of 63 MPa (RRH-1003, Yuli Electromechanical Equipment Group Co., Taizhou, China) was used to pressurize the sample.

In the setup shown in Figure 2, PSG includes a fixed linear polarizer P1 (LPNIRB100, Thorlabs Inc., Newton, NJ, USA) and a rotatable zero-order quarter-wave plate R1 (WPQ10E-633, Thorlabs Inc., Newton, NJ, USA). R1 was installed in an electric rotating stage (PRM1/MZ8, Thorlabs Inc., Newton, NJ, USA). PSA consisted of two 16-bit DoFP polarimeters (PHX050S-PC, Lucid Vision Labs Inc., Vancouver, BC, Canada, DoFP-CCD1 and DoFP-CCD2), with 2048 × 2448 pixels and 21 frames per second. Each DoFP polarimeter is capable of obtaining images in four linear polarization channels of 0°, 45°, 90° and 135° in a single measurement. Two DoFP polarimeters were installed at the transmission and reflection ends of a 50:50 non-polarizing beam splitter prism (CCM1-BS013/M, Thorlabs Inc., Newton, NJ, USA), and a fixed zero-order quarter-wave plate R2 (WPQ10E-633, Thorlabs Inc., Newton, NJ, USA) was installed between the transmission end of the prism and DoFP-CCD1.

To improve the measurement accuracy and reduce the instantaneous field-of-view (IFOV) error, a calibration strategy was used to calibrate the Mueller matrix image measurement of the setup [30]. Firstly, to reduce the error caused by the polarization direction, the extinction ratio and the intensity response of the pixels, the parallel light beam with known polarization states was used to evaluate the DoFP instrument matrix to calibrate the pixels of DoFP polarimeters. The polarization states were generated by PSG and measured by a standard polarimeter (PAX1000VIS, Thorlabs, Newton, NJ, USA). Secondly, PSA can be also calibrated by these well-calibrated DoFP polarimeters by using the same parallel beam with known polarization states as the incident light beam. In addition, a so-called PSA instrument matrix was obtained to calculate the Stokes vector image of the incident light beam from the pixel values of the two DoFP polarimeters. Thirdly, we measured a series of Stokes vector images by rotating the wave plate in PSG at angles in a given angle set, when the sample was not pressurized. Here, we considered the air as the standard, whose Mueller matrix was the unit matrix. Up to this point, we obtained a controllable PSG and a qualified PSA. After the calibration, the error of measured Mueller matrix elements normalized by $m_{11}$ was less than 0.005.

After the sample was loaded in the setup, we illuminated the sample with the parallel light beam with the known polarization states and, accordingly, recorded the Stokes vector image of the beam after the sample. Since the setup can measure the Mueller matrix with very low error after the above calibration by the air, we can calculate the Mueller matrix of the sample using Equation (1). However, the other error, excluding those in the polarization measurement, such as the image distortion from the deformed sample, may do harm to the measurement of the Mueller matrix of the sample. As a result, we focused much effort to correct the image distortion, in order to obtain the accurate Mueller matrix of the sample under different pressure values.

During the measurement, we optimized the angle set by considering both the small condition number to suppress the error accumulation and the fast measurement speed of the system. Currently, four different angles, −45°, 45°, −19.6° and 19.6°, of the rotating wave plate in PSG were used in a single measurement [31].

In order to ensure that the light beam can pass entirely through the sample, the pressurization area on the sample was a ring whose inner diameter was 22 mm, larger than the latter face of the sample. Ideally, according to the pressure conversion formula, the transfer of force without loss can be expressed as Equation (2),

(2) $$F=P_{A\;}\cdot S_{window}=P\cdot S_{jack}$$

where $P_{A}$ is the ideal equivalent pressure, $S_{window}$ is the pressurization area of the window, P is the pressure displayed on the jack dashboard [32], and $S_{jack}$(=175.84 cm^2^) is the pressurization area of the jack, which was obtained by asking the company. The transfer efficiency η (=0.8) was introduced considering the transfer loss in the actual situation. The actual equivalent pressure,$\;P_{A}^{\prime }$ on the sample can be calculated by Equation (3) from the pressure value read from the jack,

(3) $$P_{A}^{\prime }=\eta \cdot \frac{P\cdot S_{jack}}{S_{window}}\approx 3\cdot P$$

This means that when the jack operated between 0 and 60 MPa, the actual pressure on the window was about 0–180 MPa, and in the following context, we mention the pressure as the actual pressure. In the pressurization experiment, we took 12 MPa as the step pressure to carry out the experiments. Besides, we also investigated the full recovery of the sample after pressurization and monitored the change of polarization parameters in this process, so as to find the parameters that can effectively characterize the stress change of the sample.

### 2.3. Image Distortion Correction Method

During the experiment, we found that with the increase in pressure, the sample was deformed, which caused the parallel light beam to experience a certain level of distortion. The pixel-level correspondence between the incident and output light beam was destroyed, which decreased the accuracy of the Mueller matrix imaging. Additionally, the correspondence between the measurement images in different pressures was damaged, which caused harm to the stress characterization of the polarization parameters. Here, we used a correction method to correct the distortion of these images after pressurization.

In order to correct the image distortion accurately, the grid auxiliary line was drawn by a dark soft brush on the latter face of the sample, whose correspondence with the covered areas on the latter surface was physically unchanged, regardless of how the distortion occurred. Then, a manual feature point extraction method was used to correct the image distortion under different pressures according to the grid auxiliary line. The key point of the method was to select the feature points in the original image without pressure and the distorted image under certain pressure, and then obtain each feature point’s pixel coordinate index. For the example of the *i* th feature points, these were $\left(x_{0}^{i},y_{0}^{i}\right)$ and $\left(x_{1}^{i},y_{1}^{i}\right)$. If we obtained entirely *n* feature points, we obtained $x_{0}$ as an *n ×* 1 vector formed by $x_{0}^{i}$, (*i* = 1, 2, …, *n)*, and similarly, we obtained $y_{0}$, $x_{1}$, and $y_{1}$. After that, we carried out the projection transformation with a 3 × 3 transformation matrix T, that is, $\left(x_{0},y_{0},u\right)=\left(x_{1},y_{1},u\right)*T$, where u is an *n ×* 1 vector formed by 1. Three columns in T respectively represent the transformation of the image in the *x*, *y* and *z* directions. For the two-dimensional transformation shown in this case, the data in the third column will be [0, 0, 1]. *T* is specified by pairs of feature points. By substituting *T* into the *imwarp* function, we obtained the corrected image [33].

**Figure 3:**
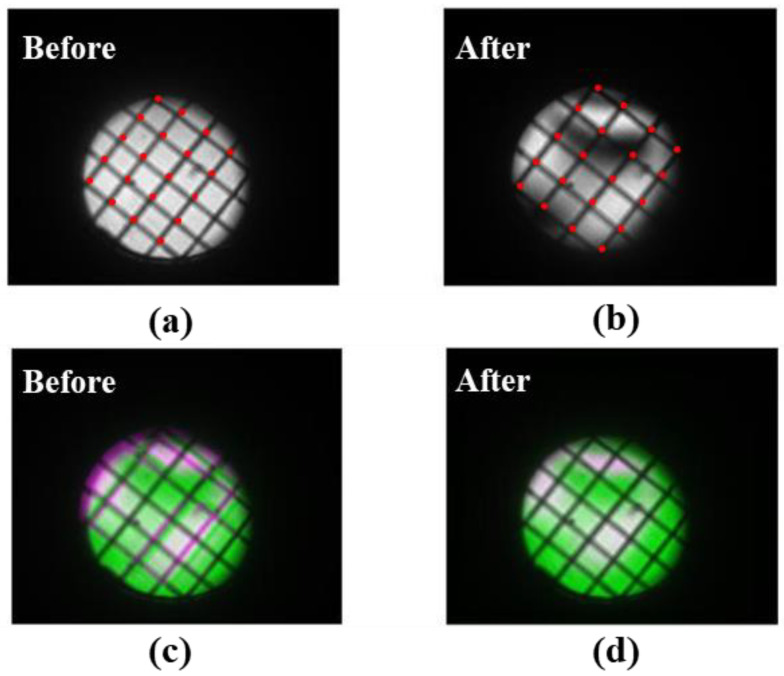
Results of the correction method. (**a**) Original image without pressure; (**b**) distorted images with 120 MPa pressure; (**c**) merged image of (**a**,**b**) with false-color algorithm; (**d**) merged image of original and corrected images with false-color algorithm. Red dots in (**a**,**b**) are the feature points.

We compared the images before and after pressure, and the raw intensity images from the 0° linear polarization channel of DoFP-CCD1 were considered as examples. Figure 3a,b respectively show the images before and after loading 120 MPa, which are marked by red dots. We can see that the distortion is very serious, and the grids in Figure 3b are slightly larger than those in Figure 3a, which may originate from the bulge of the latter face of the sample in the pressurization process. We merged these two images together with a false-color algorithm and show it in Figure 3c where the green part is the original image and the pink part is the 120 MPa pressure image. One can see that the overlap between pink and green is very severe, and the ghosts of the grid auxiliary lines are rather obvious.

We selected the feature points (marked as red points in Figure 3a,b) according to the grid auxiliary line, and then obtained the transformation matrix to correct the distorted image to the corrected image. Figure 3d shows the merged image of the original and corrected images. The pixel correspondence greatly improved after the algorithm, as the pink image was almost completely covered by the green image. The pink part that is not completely covered may have been caused by the inaccuracy of T, due to the insufficiently dense selection of feature points.

To verify the stability of this method, we manually marked the same data six times to test the error introduced by the transformation matrix due to the manual mark of feature points. Each mark process selected more than 20 pairs of feature points, and Table 2 collects and shows the nine transformation matrix elements $t_{11}\sim t_{33}$ calculated in the six marking processes. By calculating the Mean ± Var values of each element, we evaluated the influence of the manual mark on T. $t_{13},t_{23}$ were close to 0,$\;t_{33}$ is 1, which means a change of the image in the *z*-direction did not occur.

In order to verify the influence of the standard deviations of $t_{31}\;\mathrm{and}\;t_{32}$ on the pixel movement, we set all other elements as their own average values but allowed $t_{31}\;\mathrm{and}\;t_{32}$ to change within their own standard deviations, with which we constructed T accordingly. The corrected image can be calculated for each new setting of $t_{31}\;\mathrm{and}\;t_{32}$, and the pixel positions are found to change within 4 pixels. Compared with the 2048 × 2448 image, the relative error of the pixel position is less than 0.2%. Generally, from Table 2, the difference between six independent markings is so small that it indicates the above correction method is not influenced by the manual point marking.

## 3. Results

### 3.1. Characterization of Sample Changes during Pressurization and Recovery Stages by Polarization Parameters

**Figure 4:**
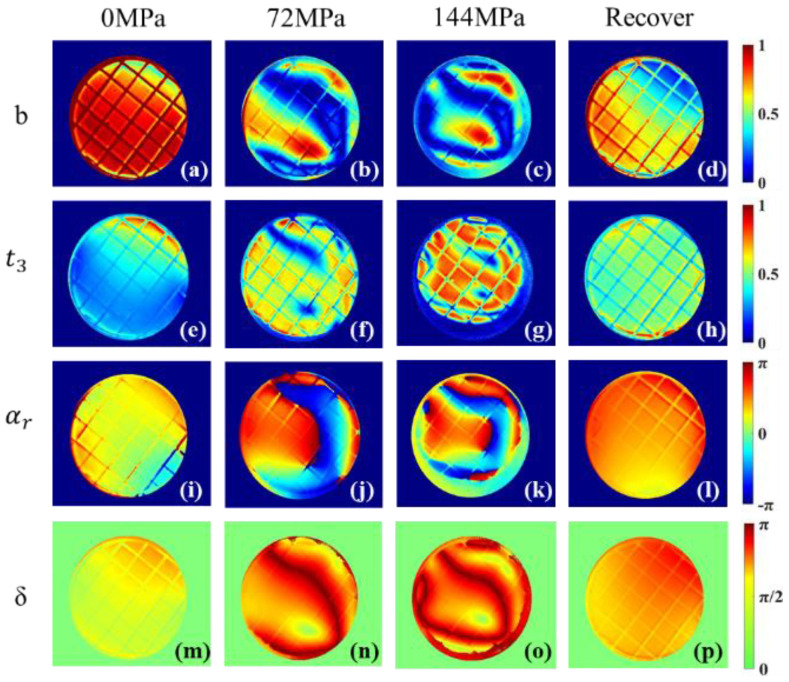
*b*, $t_{3}$,$\;\alpha_{r}$, δ  images of sample after 0 MPa, 72 MPa and 144 MPa pressure and those after 24 h recovery. (**a**–**d**): *b* images; (**e**–**h**): $t_{3}$ images; (**i**–**l**):$\;\alpha_{r}$ images; (**m**–**p**): δ images.

The results of several polarization parameters on the sample at pressurization and recovery stages are shown in Figure 4, which are *b*, $t_{3}$, $\alpha_{r}$ in the Mueller matrix transformation (MMT) technique [34] and parameter *δ* in the Mueller matrix polar decomposition (MMPD) method [35]. Their calculation formulas are shown in Equations (4)–(12), where R is the total retardance, $\varepsilon_{ijk}$ is the Levi–Cività permutation symbol, $\delta_{ij}$ is the Kronecker delta, φ is the optical rotation of magnitude. The previous literature shows that *b* is reversely related to depolarization, $t_{3}$ and *δ* are related to linear retardation, while $\alpha_{r}$ is related to the medium anisotropy [36].

(4) $$b=\frac{1}{2}\left(m_{22}+m_{33}\right),$$

(5) $$t_{3}=\sqrt{m_{42}^{2}+m_{43}^{2}},$$

(6) $$\alpha_{r}=\frac{1}{2}\arctan\left(\frac{m_{24}}{-m_{34}}\right),$$

(7) $$\alpha_{q}=\frac{1}{2}\arctan\left(\frac{-m_{24}}{m_{34}}\right),$$

(8) $$M_{R}=\left[\begin{array}{cc} 1 & \begin{array}{ccc} 0 & 0 & 0 \end{array}\\ \begin{array}{c} 0\\ 0\\ 0 \end{array} & m_{R} \end{array}\right]=\left[\begin{array}{cc} 1 & \begin{array}{ccc} 0 & 0 & 0 \end{array}\\ \begin{array}{c} 0\\ 0\\ 0 \end{array} & m_{LR} \end{array}\right]\left(\begin{array}{cc} \begin{array}{cc} 1 & 0\\ 0 & \cos2\varphi \end{array} & \begin{array}{cc} 0 & 0\\ \sin2\varphi & 0 \end{array}\\ \begin{array}{cc} 0 & -\sin2\varphi \\ 0 & 0 \end{array} & \begin{array}{cc} \cos2\varphi & 0\\ 0 & 1 \end{array} \end{array}\right)=M_{LR}M_{CR},$$

(9) $${\left(m_{R}\right)}_{ij}=\delta_{ij}\cos R+a_{i}a_{j}\left(1-\cos R\right)+\sum_{k=1}^{3}\varepsilon_{ijk}a_{k}\sin R,i,j=1,2,3$$

(10) $$\delta =\arccos\left(\sqrt{{\left(m_{R22}+m_{R33}\right)}^{2}+{\left(m_{R32}-m_{R23}\right)}^{2}}-1\right),$$

(11) $$R=\arccos\left[\frac{tr\left(M_{R}\right)}{2}-1\right]$$

(12) $$a_{i}=\frac{1}{2\sin R}\overset{3}{\underset{\;i,j=1}{\sum }}\varepsilon_{ijk}{\left(m_{R}\right)}_{jk}\;$$

We selected the above four polarization parameters to compare the sample’s images after 0 MPa, 72 MPa and 144 MPa and images after the 24 h recovery. In the horizontal direction, the polarization parameters can similarly characterize the changes in pressurization and recovery stages. When the pressure is 0 MPa, the first column images were relatively uniform and *b* in Figure 4a is close to 1 uniformly, which indicates that the sample’s depolarization was weak initially.$\;t_{3}$ in Figure 4e, together with *δ* in Figure 4m, reflect that the sample without pressure has relatively small variation in the linear retardation. Figure 4i shows the sample has an almost uniform anisotropic structure, except for its peripheries when the pressure is 0 MPa.

The jack exerts ring-shaped pressure on the sample, and the center of the sample is the clear aperture, so there is a stress transfer from the periphery to the center of the sample. When the pressure reaches 72 MPa, for the second column of Figure 4, the central areas become heterogeneous, and there are also annular structures close to the periphery. It can be inferred that at this annular area, there is a strong depolarization effect because of the small *b*, and a large retardation difference because of the dramatic changes of $t_{3}$,$\;\alpha_{r}\;\mathrm{and}\;\delta$. When the sample is pressurized to 144 MPa, a clear and complete ring-shaped area is observed in the third column of Figure 4, where the ring contracts more toward the central area than what happens at 72 MPa. Note that the values of $\alpha_{r}$ and δ obtained by Equations (6) and (10) may be wrapped, and the retardance of the sample may be larger than those values shown in the third and fourth row of Figure 4 [37]. However, we can see $\alpha_{r}$ and δ images become more heterogeneous due to the larger pressures. This indicates that the polarization parameters can characterize the process of stress transfer inside the sample during the pressurization stage. When the sample is recovered after 24 h, the fourth column of Figure 4 returns to a certain homogeneity, which is similar to that in the first column, but the values of the parameters are quite different from the initial ones. This indicates that after pressurizing to 144 MPa, the sample experiences changes in its internal structure that cannot be fully recovered in 24 h, and the proposed polarization parameters are sensitive to these changes.

### 3.2. Characterization of Elastic-Plastic Transformation of Samples Described by Polarization Parameters

In the previous experiments, we found the polarization parameters could not return to the initial state after pressurizing 144 MPa. In order to investigate the elastic–plastic transformation process in the sample, we design the experiment and chose the MMPD parameter *γ* to characterize the elastic–plastic transformation of the sample. Previous work shows that *γ* can perfectly reveal the sample’s fast axis orientation [38]. The calculation formula of *γ* is shown by Equations (13) and (14), with a magnitude of linear retardance β:

(13) $$\gamma =\frac{1}{2}\arctan(\frac{r2}{r1}),$$

(14) $$r_{i}=\frac{1}{2\sin\beta }\times \overset{3}{\underset{\;i,j=1}{\sum }}\varepsilon_{ijk}{\left(m_{LR}\right)}_{jk}\;$$

**Figure 5:**
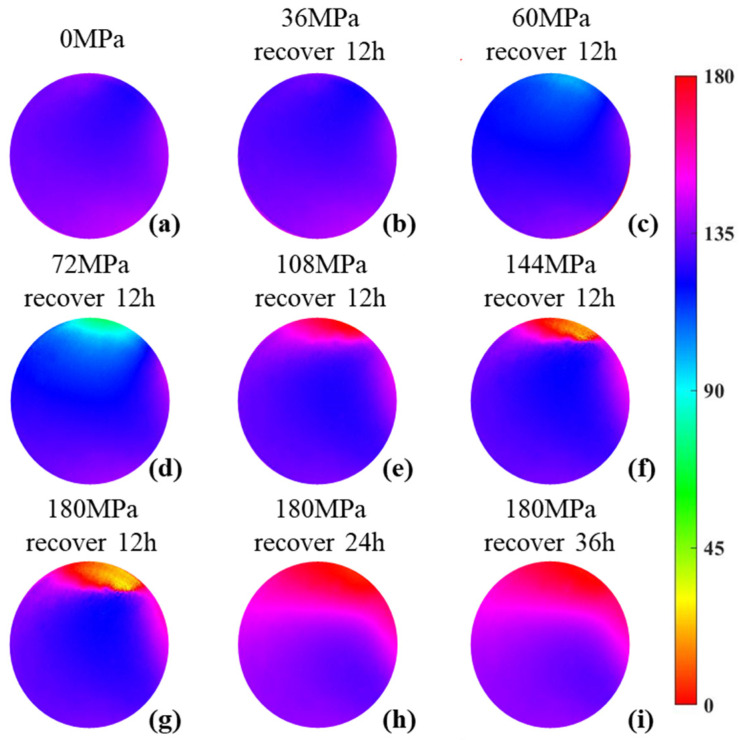
*γ* images under different pressures and recovery times. (**a**–**f**): *γ* images under different pressures; (**g**–**i**): *γ* images under different recovery times.

We carried out the experiments under different pressures and recovery times, and the results are presented in Figure 5, which shows the ability of polarization parameter to characterize the elastic–plastic transformation of the sample. Figure 5a shows the sample’s initial image of *γ*, whose homogeneity indicates that the sample is an anisotropic material with a homogeneous orientation. Figure 5b–d shows the *γ* images of the sample after 12 h recovery from being pressurized to 36 MPa, 60 MPa and 72 MPa, respectively. With the increase in pressurization pressure, *γ* gradually changes and the overall homogeneity decreases. Furthermore, since *γ* is an angle parameter with a cycle of 180 degrees, the cross-cycle variation first appears at the top right corner of Figure 5e, which means that *γ* changed so dramatically that it skipped the current cycle. Additionally, the area of the cross-cycle parts in Figure 5f,g increases continuously with the increasing pressurization pressure. Combined with the structural characteristics of the sample, the images in Figure 5 indicate that *γ*’s distributions are rather different after 12 h recovery from different pressures, and especially at the top right corners, *γ* experiences entirely different changes from the other parts. These factors imply that *γ* is sensitive to the mechanical structure behavior of the sample under pressurization.

In addition, the experimental results reveal that the sample’s recovery is closely related to the recovery time. When the maximum pressure rises to 180 MPa, the sample obviously cannot fully recover within 12 h, so we extended the recovery time. Figure 5h,i shows *γ* images after 24 h and 36 h recovery, respectively. If *γ* no longer changes significantly, the sample is considered to have reached a stable state, and the degree of unevenness in *γ* may be used as an indicator for the recovery degree of the sample.

**Figure 6:**
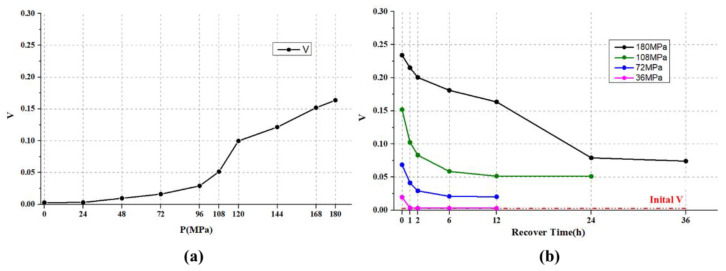
(**a**) *V* for different pressurization pressures; (**b**) *V* for different recovery times.

In order to quantitatively describe this process, we define the circular standard deviation of the γ images as *V*. The *V* values after 12 h recovery from different pressurization values are collected and shown in Figure 6a. From Figure 6a, *V* continues to increase with the increase in pressurization pressure, and the slope increases sharply between 96 MPa and 120 MPa, which may be the critical pressure range where the elastic–plastic transition occurs.

Meanwhile, *V* values’ temporal changes during the sample’s recovery process under different pressurization pressures are shown in Figure 6b. Note that the initial *V* value is estimated when there is no pressure applied to the sample. Under low pressures, *V* quickly drops to the initial *V* values. With the increase in pressure, the time for *V* to reach the stable value increases, and the stable *V* value gradually deviates from the initial *V*. Under pressure of 180 MPa, *V* decreases continuously within 24 h after depressurization to 0 MPa and reaches its stable value after 36 h. This means that the sample may recover to its stable state after 36 h recovery, but it cannot return to the initial state, which indicates that the sample has irreversible plastic deformation during the pressurization process. In this sense, we are able to draw the conclusion that *V* can effectively characterize the elastic–plastic transformation of the sample under pressure.

### 3.3. Two-Layered Wave Plate Simulation

In the loading experiment, we find a difference between the values of $\alpha_{q}$ and $\alpha_{r}$ calculated by Equations (6) and (7), indicating that the sample may have a multilayer structure [39]. In order to describe the structural stratification of this sample more accurately, we build a two-layered wave plate model to describe the polarization properties of the sample and carry out the analysis according to the existing experimental results. In the model, the polarization property of the sample can be approximated to the successiveness of two optical retardation components because the dichroism and depolarization properties of the sample can be neglected under an unpressurized state.

The Mueller matrix of one wave plate can be expressed as Equation (15), where θ is the orientation of the fast axis of this wave plate and δ is the retardance [33]. The Mueller matrix of a two-layered wave plate model can be expressed as Equation (16), as the light passes through $M_{LR1}$ and then through $M_{LR2}$. $\theta_{1}$ and $\delta_{1}\;$are the fast axis orientation and the retardance of the first wave plate, respectively, and $\theta_{2}$ and $\delta_{2}\;$are those of the second wave plate.

(15) $$M_{LR}=\left(\begin{array}{cc} \begin{array}{cc} 1 & 0\\ 0 & \cos^{2}2\theta +\sin^{2}2\theta \cos\delta \end{array} & \begin{array}{cc} 0 & 0\\ \sin2\theta \cos2\theta \left(1-\cos\delta \right) & -\sin2\theta \sin\delta \end{array}\\ \begin{array}{cc} 0 & \sin2\theta \cos2\theta \left(1-\cos\delta \right)\\ 0 & \sin2\theta \sin\delta \end{array} & \begin{array}{cc} \sin^{2}2\theta +\cos^{2}2\theta \cos\delta & \cos2\theta \sin\delta \\ -\cos2\theta \sin\delta & \cos\delta \end{array} \end{array}\right),$$

(16) $$M_{LR2}M_{LR1}=\left(\begin{array}{cccc} 1 & 0 & 0 & 0\\ 0 & \cos2\theta_{2} & -\sin2\theta_{2} & 0\\ 0 & \sin2\theta_{2} & \cos2\theta_{2} & 0\\ 0 & 0 & 0 & 1 \end{array}\right)\left(\begin{array}{cccc} 1 & 0 & 0 & 0\\ 0 & 1 & 0 & 0\\ 0 & 0 & \cos\left(\delta_{1}+\delta_{2}\right) & \sin\left(\delta_{1}+\delta_{2}\right)\\ 0 & 0 & -\sin\left(\delta_{1}+\delta_{2}\right) & \cos\left(\delta_{1}+\delta_{2}\right) \end{array}\right)\left(\begin{array}{cccc} 1 & 0 & 0 & 0\\ 0 & \cos2\theta_{1} & \sin2\theta_{1} & 0\\ 0 & -\sin2\theta_{1} & \cos2\theta_{1} & 0\\ 0 & 0 & 0 & 1 \end{array}\right)$$

Then, the Mueller matrix of the sample is deliberately considered to be equivalent to the Mueller matrix defined as Equation (16). The two-layered wave plate model introduces four parameters, $\theta_{1},\theta_{2},\delta_{1},\delta_{2}$. We expect to solve these four parameters according to the measured Mueller matrix.

**Figure 7:**
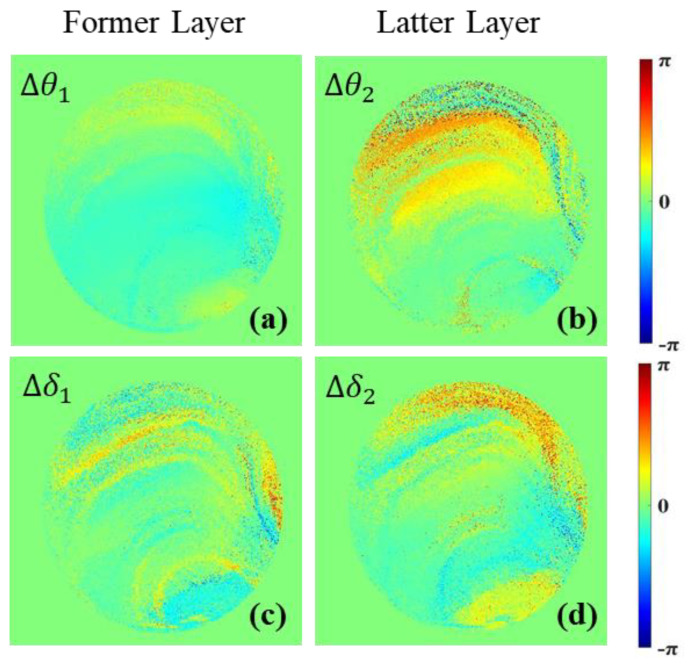
Two-layered difference of retardance parameters $\Delta \theta_{1},\Delta \theta_{2},\Delta \delta_{1},\Delta \delta_{2}$. (**a**)$\;\Delta \theta_{1}\;$ image; (**b**) $\Delta \theta_{2}\;$ image; (**c**) $\Delta \delta_{1}\;$ image; (**d**)$\;\Delta \delta_{2}$ image.

The initial $\theta_{1-1},\theta_{2-1},\delta_{1-1},\delta_{2-1}$ are solved by the initial Mueller matrix of the sample, which is measured before the loading pressure, and the final $\theta_{1-2},\theta_{2-2},\delta_{1-2},\delta_{2-2}$ are obtained by the Mueller matrix fully recovered after being loaded to 180 MPa. Then, $\Delta \theta_{1}\left(\equiv \theta_{1-2}-\theta_{1-1}\right),\;\Delta \theta_{2}\left(\equiv \theta_{2-2}-\theta_{2-1}\right),\Delta \delta_{1}\left(\equiv \delta_{1-2}-\delta_{1-1}\right),\Delta \delta_{2}\left(\equiv \delta_{2-2}-\delta_{2-1}\right)$ are obtained, as shown in Figure 7. Figure 7a,c shows the parameter differences $\Delta \theta_{1},\Delta \delta_{1}$ of the former layer, respectively, and Figure 7b,d shows the parameter differences $\Delta \theta_{2},\Delta \delta_{2}$ of the latter layer, respectively. It is obvious that $\Delta \theta_{2}$ in the latter layer is significantly greater than $\Delta \theta_{1}$ in the former layer. Both $\Delta \delta_{1}$ and $\Delta \delta_{2}$ distribute unevenly and their values are large, but the difference between them is not quite noticeable. This means that, relatively, θ is sensitive to the layers but δ is sensitive to the pressurization pressure.

The part of the sample near the former face is equivalent to the former layer wave plate, and the part of the latter face is equivalent to the latter layer wave plate. As seen in Figure 7, in the process of pressurization and recovery, the change of the latter layer is more significant than that of the former layer. In addition, the texture structure appears in the upper half of each image of Figure 7, which may be the trail of stress transfer during the stage of pressurization.

**Figure 8:**
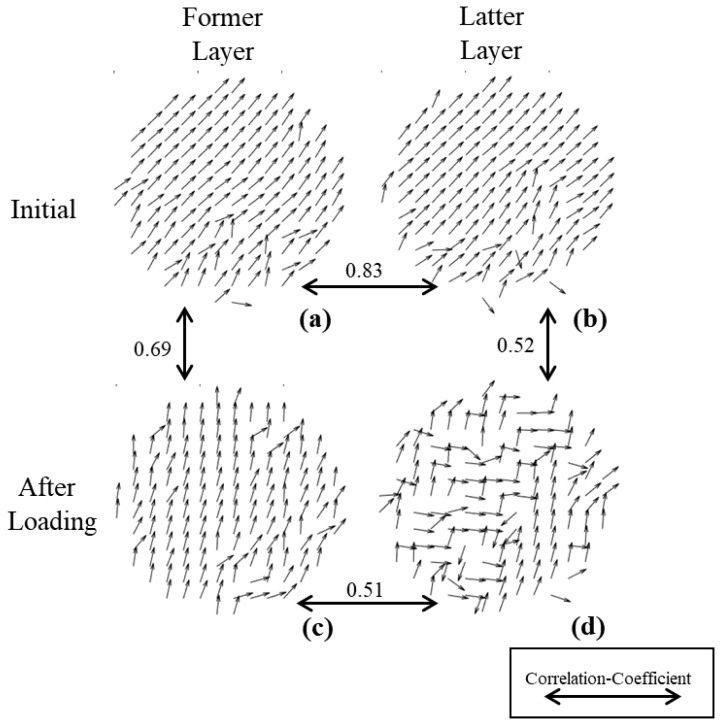
Fast axis orientation flow diagram of two-layered simulation. (**a**) initial image of former layer; (**b**) initial image of latter layer; (**c**) after loading image of former layer; (**d**) after loading image of latter layer.

In order to explore the different reactions of the two-layered model before and after pressurization, we draw θ images in Figure 8. Figure 8a,b shows the θ images of the former and latter layers of the sample before pressurization, and Figure 8c,d represents those after 24 h recovery. In order to be more intuitive, we calculate the correlation coefficients between the matrices respectively, which are indicated on the two-way arrows.

From the horizontal direction, the value of θ for the two layers before pressurization is highly consistent, and the correlation coefficient is 0.83. After recovery, the value of θ of the two layers change in a disorderly manner, and the correlation coefficient decreased to 0.51. This indicates that the loading-pressure stage causes irreversible damage to the sample, and the internal structure changes, which echoes the conclusions drawn in Figure 4.

From the vertical direction, Figure 8c shows that the former layer still maintains good θ homogeneity after being pressurized, and the correlation coefficient with the initial image in Figure 8a is 0.69. Figure 8d shows that θ of the latter layer is disordered after being pressurized, and the correlation coefficient decreases to 0.52 with the initial image Figure 8b. This shows that in the pressurization, the latter layer undergoes a greater change than the former layer.

## 4. Discussion

### 4.1. Finite Element Analysis of Sample after Pressurization

In order to evaluate the accuracy and effectiveness of the polarization parameters, the finite element simulation of the stress inside the sample is carried out according to the actual experimental situation by Solidworks (2020, educational trial version, Dassault Systèmes SolidWorks Corporation, Concord, CA, USA). The model is built according to the actual sample size, and the interaction mode between the model and the base is set as sliding friction while the finite element parameters of the sample and base are collected and shown in Table 3.

**Figure 9:**
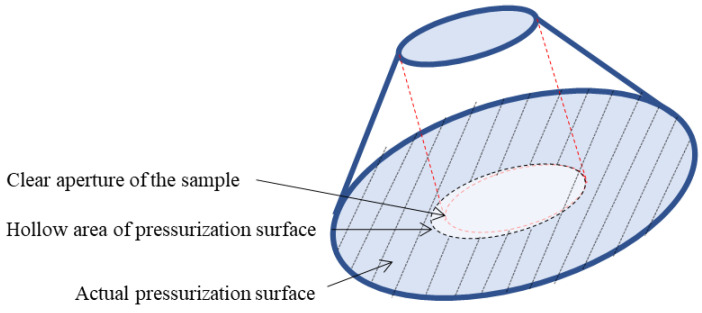
Relationship of the clear aperture of the sample with the pressurization surface.

**Figure 10:**
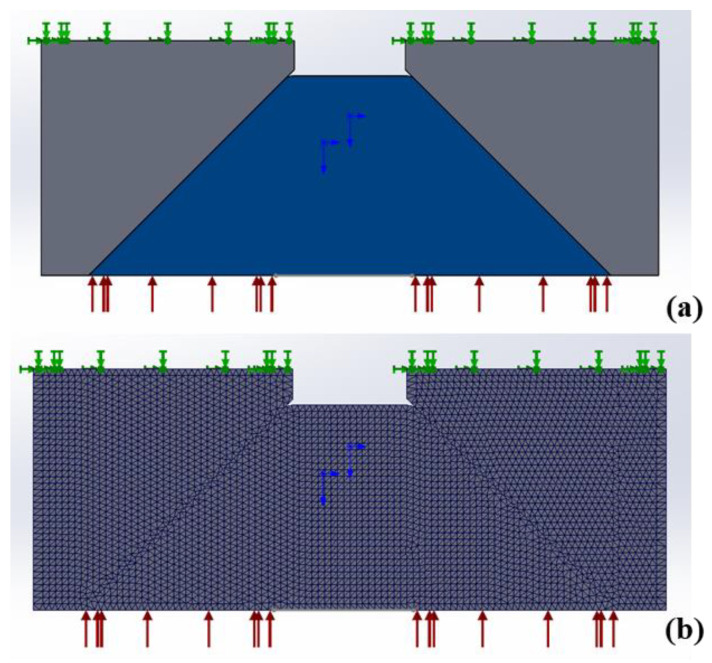
Boundary constraint and mesh division of finite element model. (**a**): boundary constraint image; (**b**): mesh division image.

There is some mismatch between the central ring-shaped pressurization surface and the clear aperture of the sample due to the machining errors of the mounting bracket, as shown in Figure 9. Note that the hollow area of the pressurization surface entirely covers the clear aperture of the sample. When pressure is applied to the sample, the mismatched offset of the pressure center is set to be 2 mm, and the boundary constraint distribution and mesh element division of the finite element model are shown in Figure 10.

**Figure 11:**
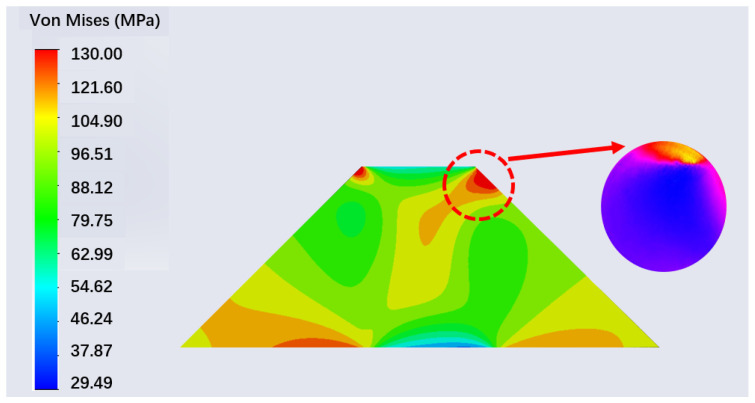
Mises stress diagram of sample.

Figure 11 shows the Mises stress of the sectional sample under 108 MPa. Due to the offset of the annular pressure area, the internal stress of the sample is not symmetrical, and the stress near the latter periphery on the offset direction is larger than that on the opposite side. With the pressure increasing, the stress inside the sample tends to accumulate on this side. This conclusion can correspond to the phenomenon of Figure 5, explaining why the experimental results change unevenly.

**Figure 12:**
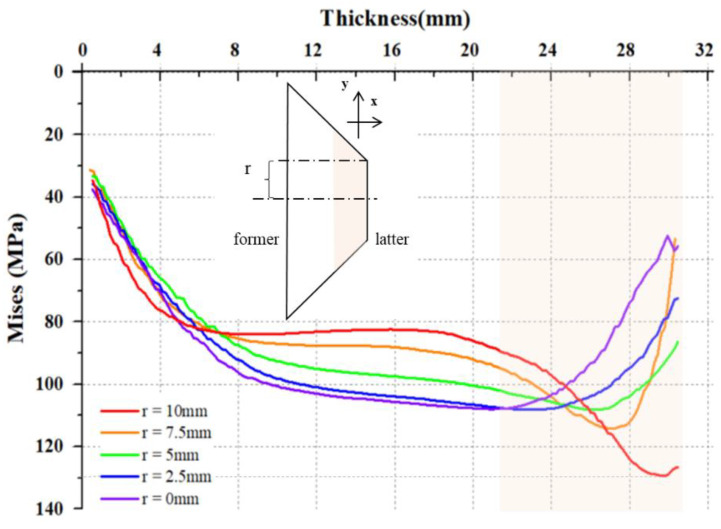
The stress along *x*-axis of sample as different distances to the central line.

The analytical results of the two-layered wave plate in Section 3.3 show that after pressurization, the sample’s polarization properties can be described as a two-layered model. In order to further understand this result, we especially plot several stress curves inside the sample according to Figure 11. Figure 12 shows the Mises stress changes along the x-axis direction (light propagating direction) when a pressure of 108 MPa is applied to the sample, while the y-axis direction is also determined. We define a value, *r*, as the distance to the central line along the y-axis, and specifically, take the side with strong stress accumulation as the example. For the plot axes in Figure 12, the top horizontal axis represents the thickness of the sample and the left vertical axis represents the Mises stress. The five curves in Figure 12 represent the stress varying with different *r*.

In Figure 12, the orange-shadow areas are accordingly marked in both the model and the plot to show the possible range of the latter layer, and the other parts are considered as the former layer. From the five selected curves in Figure 12, the stress consistency of the latter layer of the sample is far less than that of the former layer, and the maximum stress appears in the curve of the latter layer with *r* = 10 mm, which corresponds to the position of the sample near the periphery in the actual structure. The maximum stress value is around 130 MPa, exceeding the yield limit of the material, which means that plastic deformation has been produced at the periphery of the sample. These prove that, firstly, with different thicknesses, the stress accumulation inside the sample is unevenly distributed, and the largest value appears at the periphery part of the sample. Meanwhile, one can see that in the latter layer, the values of curves are rather different, which means that the stress change at this layer is striking at both *x* and *y* directions when pressurizing a sample. Secondly, both the experimental and simulation results reveal the critical pressure of the elastic–plastic transformation. In Figure 6, we see that the possible critical pressure for the elastic–plastic transition of the sample appears between 96 and 120 MPa, as the slope of *V* increases significantly in this zone. As this happens, the finite element simulation, with a 108 MPa pressurization surface, results in stress accumulation in the periphery of the sample and reaches the yield limit, which triggers the plastic deformation in this area. So, the results of simulation and experiment can be understood as corresponding. Besides that, we prove that *V*, the degree of unevenness in *γ*, is able to characterize the elastic–plastic transformation of the material.

According to Figure 12, it seems as though a multilayer model would be more reasonable. However, it has been proved in the literature that multilayer wave plates can be equivalently represented in the form of two-layered wave plates [40], such that the two-layered model in the work is the simplest but most effective for the sample. However, to accurately describe and analyze the internal stress structure of the sample, the detailed multilayer model or tomographic method should be introduced to interpret or measure the stress properties of this kind of sample.

### 4.2. Characterization Potential of Other Parameters

**Figure 13:**
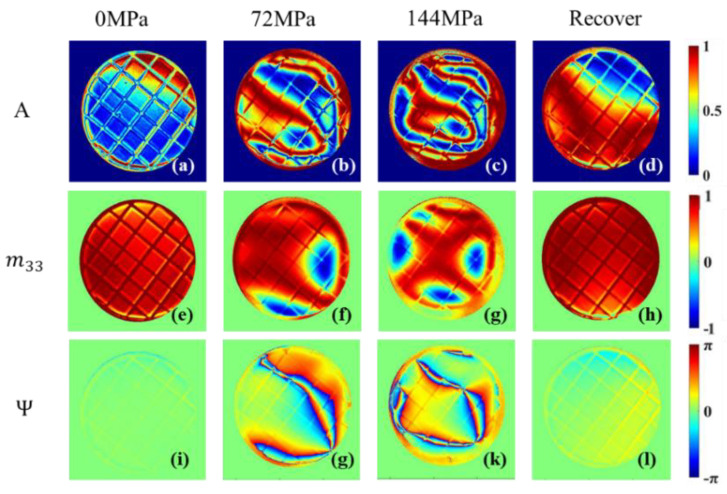
*A*,$\;m_{33},\;\Psi$ images of sample at 0 MPa, 72 MPa, 144 MPa and images after 24 h recovery. (**a**–**d**): *A* images; (**e**–**h**): $m_{33}$ images; (**i**–**l**): Ψ images.

In addition to the results described above, we also find that many other parameters are also sensitive to the pressurization process of the sample, as shown in Figure 13. The calculation formulas of *A* and Ψ parameters are given in Equations (17)–(19).

(17) $$A=2bt_{1}/\left(b^{2}+t_{1}{}^{2}\right),$$

(18) $$t_{1}=\frac{1}{2}\sqrt{{\left(m_{22}-m_{33}\right)}^{2}+{\left(m_{23}+m_{32}\right)}^{2}},$$

(19) $$\Psi =\frac{1}{2}\arctan\left[\left(M_{R21}-M_{R12}\right)/\left(M_{R11}+M_{R22}\right)\right]$$

Figure 13 shows that *A* and Ψ, calculated by Equations (17) and (19), can show the ring-shaped area under the loading stage, which is similar to those parameters in Figure 4. However, the boundaries of the ring-shaped area are different due to their different physical meanings, which implies more specific meanings carried by these separate parameters than those in Figure 4. Meanwhile, many other parameters also deserve our attention. They may be related to the individual differences between the samples, and may also contain more mechanical information about the material.

The results in this work encourage us to believe that polarization parameters have great research potential for characterizing the stress change of materials. Note that the annular pressurization method is currently adopted in this study, rather than the uniform water pressure of the observation window in the actual working environment. However, the polarization parameters proven in these experiments are still promising for comprehensively monitoring a working submersible window. Since the polarization parameters describe physical properties such as the orientation angles, retardance, and the anisotropy of the window under pressure, they have little relationship to the manner of pressurization. In the future, a real water pressure environment should be considered to characterize the internal stress changes of the observation window accordingly, and powerful polarization parameters are promising for comprehensively monitoring the working submersible’s window in the future.

On the other side, more possibilities can be imagined based on the results of this work. For example, one can directly measure the polarization parameters and does not need to measure the whole Mueller matrix. Two-dimensional imaging is not necessary and can be replaced by specified dot measurement. Additionally, according to the requirement of in situ monitoring of the submersible window, other methods can be developed to overcome the difficulties in this work such as image distortion, polarization calibration, etc.

## 5. Conclusions

Stress detection of observation windows is a key issue in the process of ensuring the safety of deep manned submersible. In this paper, we present a method based on Mueller matrix imaging and build the experimental setup to measure the stress accumulation and recovery situation inside the window. Experimental results support the idea that some polarization parameters can effectively characterize the internal stress transfer and elastic–plastic transformation of the window. Furthermore, we also use the two-layered wave plate model to analyze the internal stress structure of the window under pressurization, and the results show the difference of the former and latter layers of the window in the pressurization process. In addition, we present a finite element simulation explaining the stress accumulation at different thicknesses of the window, which verifies the effectiveness and innovation of our method in stress evaluation. Finally, more possibilities are discussed amid the target of this work by using other polarization parameters derived from Mueller matrix imaging. It is promising that this method can provide a new method of monitoring window stress, which helps to further ensure the safety of deep manned submersibles.

## Figures and Tables

**Table 1:** Mechanical parameters of the PMMA sample.

Properties	Value
Density/(g/cm^3^)	1.186
Tensile Modulus/GPa	3.13
Yield Strength/MPa	129
Poisson’s ratio	0.37
Refractive index	1.49

**Table 2:** Elements of transformation matrix for six independent markings.

Number	*t*_11_	*t*_12_	*t*_13_	*t*_21_	*t*_22_	*t*_23_	*t*_31_	*t*_32_	*t*_33_
1	0.8452	−0.0135	−1.47 × 10^−5^	0.0075	0.8699	4.45 × 10^−6^	125.6382	150.0923	1
2	0.8322	−0.0158	−2.13 × 10^−5^	0.0077	0.8613	5.34 × 10^−6^	133.1351	154.9269	1
3	0.8250	−0.0209	−2.19 × 10^−5^	0.0074	0.8470	2.20 × 10^−6^	130.6332	151.8865	1
4	0.8434	−0.0099	−1.47 × 10^−5^	0.0116	0.8678	5.71 × 10^−6^	127.6634	150.0143	1
5	0.8436	−0.0126	−1.22 × 10^−5^	0.0147	0.8818	1.16 × 10^−6^	129.1101	149.3359	1
6	0.8437	−0.0072	−1.60 × 10^−5^	−0.0026	0.8559	3.04 × 10^−6^	131.3437	152.5151	1
Mean ± var	0.8389 ± 0.0083	−0.0134 ± 0.0048	−1.44 × 10^−5^ ± 7.38 × 10^−6^	0.0077 ± 0.0059	0.8640 ± 0.0121	5.39 × 10^−6^ ± 3.32 × 10^−6^	129.5873 ± 2.6941	151.4619 ± 2.0874	1 ± 0

**Table 3:** Parameters for finite element simulation.

Properties	Value
Sample’s Density/(g/cm^3^)	1.186
Sample’s Tensile Modulus/MPa	3130
Sample’s Yield Strength/MPa	121
Sample’s Poisson’s ratio	0.37
Base’s Density/(g/cm^3^)	7.85
Base’s Tensile Modulus/MPa	200,000
Base’s Yield Strength/MPa	551
Base’s Poisson’s ratio	0.3
Friction coefficient	0.05

## Data Availability

Data sharing not applicable.
